# Impact of depressed state on attention and language processing during news broadcasts: EEG analysis and machine learning approach

**DOI:** 10.1038/s41598-022-24319-x

**Published:** 2022-11-28

**Authors:** Kohei Fuseda, Hiroki Watanabe, Atsushi Matsumoto, Junpei Saito, Yasushi Naruse, Aya S. Ihara

**Affiliations:** 1 Center for Information and Neural Networks, Advanced ICT Research Institute, National Institute of Information and Communications Technology, and Osaka University, 588-2 Iwaoka, Iwaoka-cho, Nishi-ku, Kobe, Japan; 2 Present Address: Bunkyo Gakuin University, Fujimino, Saitama, Japan; 3 Present Address: Kansai University of Welfare Sciences, Kashiwara, Osaka, Japan

**Keywords:** Attention, Language

## Abstract

While information enriches daily life, it can also sometimes have a negative impact, depending on an individual’s mental state. We recorded electroencephalogram (EEG) signals from depressed and non-depressed individuals classified based on the Beck Depression Inventory-II score while they listened to news to clarify differences in their attention to affective information and the impact of attentional bias on language processing. Results showed that depressed individuals are characterized by delayed attention to positive news and require a more increased load on language processing. The feasibility of detecting a depressed state using these EEG characteristics was evaluated by classifying individuals as depressed and non-depressed individuals. The area under the curve in the models trained by the EEG features used was 0.73. This result shows that individuals’ mental states may be assessed based on EEG measured during daily activities like listening to news.

## Introduction

In the current era of information overload, individuals receive an excessive amount of information daily, regardless of their wish to receive such news. While information enriches daily life, it can also have a negative impact such as development of stress and eventually mental illness, depending on an individual’s mental state. Negative information can influence human mental health negatively^1,2^. For example, online surveys for college students revealed that more exposure to information on COVID-19 was associated with more significant self-reported depression^3^ and anxiety^4^. To promote the comfortable and healthy use of information for everyone, it is important to assess the individual’s mental state objectively when they are receiving information. In this study, we focused on brain activity measured during the receipt of information while participants were in a depressed state. Supposing the individuals’ states of depression may be predicted from their brain activities, this work could lead to an application based on EEG that encourages behavioral change; for example, reducing the amount of time spent on exposing oneself to negative information via the Internet or social media.

Focusing on how individuals attend to the negative information may contribute to our purpose. Attentional bias for negative information (also called negativity bias)^5–11^ has been considered to contribute to the cause and maintenance of depression^12–15^. Furthermore, such attentional bias has been found in people who are at high risk of depression such as subclinically depressed individuals^16,17^, people with remitted depression^18^, and children with depressed mothers^19^. Pronounced attentional bias for negative information is due to the mood congruency effect^14,20^ which entails enhanced information processing when the affective valence of input information is congruent with the information receiver’s mood^21^. Conversely, some reports show that patients with depression^22^ and subclinically depressed individuals^17,23^ reduce the allocation of attentional resources to positive information, whereas other studies found no attentional bias for negative information in patients with depression^24–26^.

It cannot be ruled out that the inconsistent results of attentional bias for negative information may stem from the short duration of exposure to negative information. Previous studies that focus on attentional bias used traditional psychological paradigms with visual stimuli such as the emotional Stroop task^24,27–30^, Posner task^16,17^, and dot-probe task^22,25,31–33^, and examined attention to affective stimuli over a short duration (i.e., a few hundred milliseconds to a few seconds^17,23^). Koster et al.^17^ showed that negative bias occurred in the dysphoric individuals when the stimulus duration was 500 ms and 1500 ms, not when it was 250 ms. Based on these results, in this study, we aimed to investigate the attentional biases of people who have not been diagnosed with mental diseases when exposed to affective natural speech. The task of listening to natural speech enables participants to be exposed to affective stimuli for a longer duration than the traditional paradigms. Furthermore, it allows us to examine information processing in situations that are as close to everyday life as possible, rather than in paradigms designed for experiments. Thus, research using affective natural speech may further shed light on attentional biases for negative information.

In addition, our research focused on the effects of depression on language comprehension processing as depression is reported to cause difficulties in the non-affective processing of information with negative valence. For example, the reaction time to the lexical judgment of negative words was longer for depressed individuals than it was for non-depressed individuals^34^. This is interpreted by the affective interference theory, which states that prioritizing the processing of negative information causes non-affective processing to function improperly^27,34–36^. Despite this, whether depression affects the language comprehension process—especially semantic processing—remains unclear.

**Figure 1:**
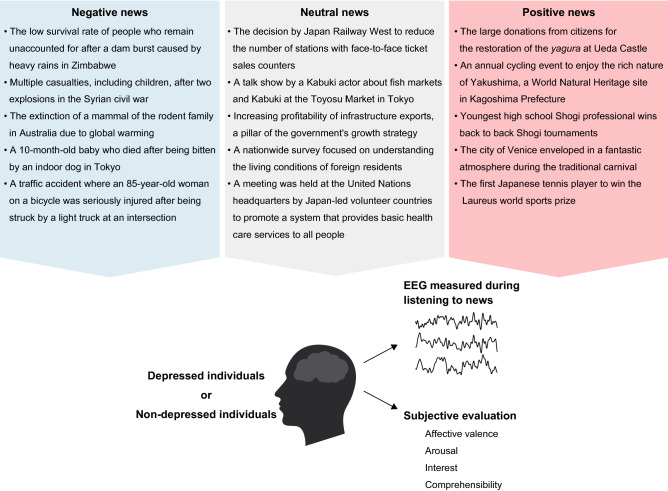
Summary of news contents played to participants during the EEG experiment and acquired data. Outlines of 15 news items (five for each positive, neutral, and negative news condition) selected are summarized at the top. In the experiment, EEG was measured while the participants were listening to the news. Immediately after, they evaluated the content on a 5-point scale: affective valence, arousal, interest, and comprehensibility.

Therefore, we adopted positive, neutral, or negative radio news broadcasted in Japan (Fig. 1, Supplementary Table S1) as stimuli and investigated whether the attention to affective information differed between depressed and non-depressed individuals who have not been diagnosed with mental diseases. In addition, how attentional bias influences language processing, especially semantic processing, was also elucidated. In this study, participants were divided into depressed individuals and non-depressed individuals based on the Japanese version of the Beck Depression Inventory (BDI)-II^37^ (Table 1). Based on the above-mentioned items, we formulated the following two hypotheses. First, the attentional bias for negative information is stronger in depressed individuals than it is in non-depressed individuals with exposure to the affective news content. Subsequently, the depressed individuals allocate more attentional resources to negative news than to positive news. Second, due to affective interference, the depressed individuals will require more load on language comprehension (semantic processing on words) while listening to negative news than non-depressed individuals.

**Table 1:** Characteristics of the depressed and non-depressed individuals.

Characteristics		Depressed	Non-depressed
Mean age		31.3 ± 10.5	33.8 ± 10.0
Sex (n)	Female	17	52
	Male	15	51
Mean LQ		70.1 ± 55.6	82.8 ± 40.2
Academic history (n)	Junior high school graduate	0	1
	High school graduate	4	14
	Junior college/vocational school student or graduate	8	11
	University student or graduate	19	66
	Graduate school student or master’s/doctoral degree holders	1	11
Mean BDI-II score		21.1 ± 8.6	6.6 ± 3.3

The values following ± indicate the standard deviations.

LQ, laterality quotient; BDI-II, Beck Depression Inventory-II.

One method of investigating the attentional biases and semantic processing on words is to observe event-related potential (ERP) components time-locked to stimuli onset by measuring electroencephalograms (EEG). For example, the N1 and P2 reflect perceptual processing^38^ and are negative and positive components that peak at approximately 100 and 200 ms after the onset of the auditory stimuli, respectively. Both responses are exogenous components elicited by the presentation of a physical stimulus. Their amplitudes are affected by the change of attentional resources allocation to the stimuli^38–40^. Thus, the changes in these responses can be used to examine the perceptual processing characteristics of depression. In contrast to these exogenous components, one of the endogenous components is N400 which is negativity that peaks at approximately 400 ms after the onset of meaningful stimuli. This component reflects semantic processing^41–43^, with amplitudes that vary based on the ease of accessing information from long-term memory and integrating semantic representations into preceding contexts^44^. Therefore, by using N1, P2, and N400 as indices, differences in perceptual and semantic processing between depressed and non-depressed individuals can be considered.

Despite the effectiveness of measuring ERP for our research purpose, it is generally difficult to observe ERP response time-locked to word onset included in natural speech like news broadcasts. Such natural speech contains only a small interval between words and, thus, EEG responses to each word are overlapped. Calculating temporal response function (TRF) weights^45^ to word onset is one solution to address this issue. TRF describes the linear mapping between ongoing stimuli (i.e., words within the news in case of news stimuli) and ongoing EEG data. Since TRF allows the separation of overlapping responses due to different stimuli close in time, this method is suitable for the analysis of EEG signals to words in natural speech. Our previous study demonstrated that the TRF weights time-locked to word onset included in natural speech showed components corresponding to N1, P2, and N400^46^. Thus, measuring these TRF components corresponding to ERP components possibly allows for quantifying the attentional resource allocation or load on semantic processing on words. To clarify the effect of depression and/or news contents on attentional bias or the load on semantic processing, we investigated group effects (depressed individuals and non-depressed individuals) and news effects (negative, neutral, and positive) on the latency and amplitude of each component.

Another advantage of using the news broadcast as stimuli over those used in the traditional paradigms was that listening to a news broadcast is a daily activity. If a depression differentiates the attentional biases or language processing, the depressed states can be predicted by simply listening to natural speech stimuli in an environment that is close to their reality. Thus, in addition to clarifying the characteristics of depressed individuals’ EEG responses to natural speech stimuli, we verified the usefulness of these responses’ applications. To this end, we evaluated the performance of classifying individuals into depression and non-depression, using EEG features and linear support vector machine (SVM), to investigate the feasibility of detecting depressed individuals.

## Results

### Subjective evaluation of news

**Figure 2:**
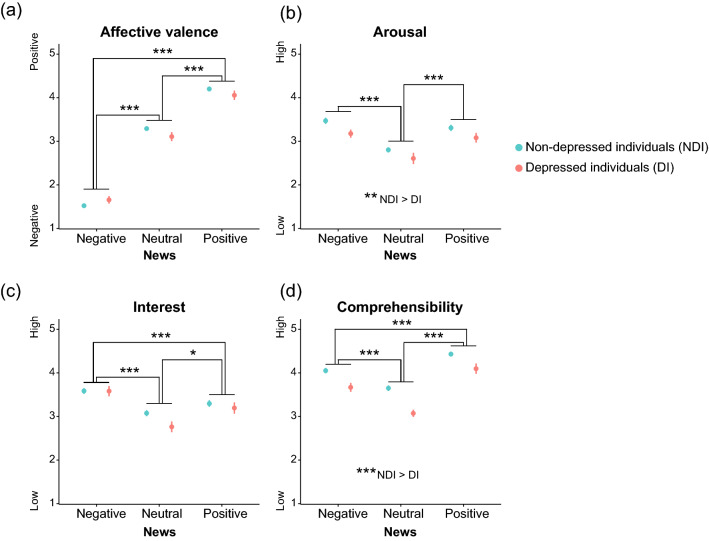
Differences in subjective evaluation between groups and between news. (**a**) Affective valence was higher for positive news than for neutral and negative news, and that for neutral news was significantly higher than that for negative news. (**b**) Arousal was higher for the negative and positive news than for the neutral news. There was no significant difference between the positive and negative news. In addition, the non-depressed individuals showed higher arousal than the depressed individuals. (**c**) Interest for the negative news was higher than that for the positive and neutral ones did, and that interest for the positive news was higher than neutral one. (**d**) Comprehensibility for the positive news was higher than those for the negative and neutral news, and comprehensibility for the negative news was higher than that for the neutral news. In addition, the news was more comprehensible for non-depressed individuals than for depressed individuals. Each circle and error bar represent the mean and standard error, respectively. *p < 0.05, **p < 0.01, and ***p < 0.005).

In the EEG experiment, participants performed subjective evaluations on each news item immediately after listening to it, based on the following items: affective valence, arousal, interest, and comprehensibility. We investigated the effect of Group (depressed and non-depressed individuals) and News (positive, neutral, and negative news) on these evaluations using a two-way mixed-design analysis of variance (ANOVA). Concordant with the preliminary survey results (Table 2; see “Materials” for details), news contents influenced affective valence, arousal, interest, and comprehensibility. First, a significant main effect of News on affective valence (*F* (2, 266) = 554.73, ε = 0.92, *p* = 9.50 × 10^–88^, η_*p*_^2^ = 0.807) was found (Fig. 2a). The affective valence increased in the following order: positive, neutral, and negative news—that for positive news was significantly higher than that for neutral (*p* = 6.18 × 10^–33^) and negative news (*p* = 1.74 × 10^–59^), and that for neutral news was significantly higher than that for negative news (*p* = 4.41 × 10^–40^).

**Table 2:** Characteristics of news selected based on the preliminary survey (N = 160).

Items	News conditions			Significant differences (Bonferroni corrected *p*-value)
	Negative	Neutral	Positive	
Affective valence	1.8 ± 0.6	3.2 ± 0.5	3.9 ± 0.7	Negative < Neutral (*p* = 1.01 × 10^–44^) Neutral < Positive (*p* = 4.09 × 10^–55^) Negative < Positive (*p* = 2.13 × 10^–27^)
Arousal	3.1 ± 0.7	2.8 ± 0.6	3.0 ± 0.7	Neutral < Negative (*p* = 2.17 × 10^–11^) Neutral < Positive (*p* = 4.89 × 10^–10^)
Interest	3.1 ± 0.8	2.7 ± 0.7	2.8 ± 0.8	Neutral < Negative (*p* = 3.28 × 10^–12^) Positive < Negative (*p* = 1.02 × 10^–4^) Neutral < Positive (*p* = 0.039)
Comprehensibility	3.7 ± 0.8	3.4 ± 0.7	3.9 ± 0.7	Neutral < Negative (*p* = 1.18 × 10^–8^) Negative < Positive (*p* = 1.87 × 10^–7^) Neutral < Positive (*p* = 1.02 × 10^–20^)

Mean ± the standard deviations.

Second, the main effect of News on arousal was significant (*F* (2, 266) = 27.02, ε = 0.95, *p* = 6.57 × 10^–11^, η_*p*_^2^ = 0.169) (Fig. 2b), indicating that the negative and positive news showed significantly higher arousal than the neutral ones did (*p* = 2.35 × 10^–10^ and *p* = 1.97 × 10^–8^, respectively). Importantly, for our experiment that focused on the attentional bias regarding negative information, no significant difference was noted between the positive and negative news (*p* = 0.697). In addition, a significant main effect of Group was found (*F* (1, 133) = 7.79, *p* = 0.006, η_*p*_^2^ = 0.055): arousal in the non-depressed individuals was significantly higher than that in depressed individuals. The subjective evaluation results showed that negative news was evaluated as negative content and positive news as positive content, confirming the validity of the news conditions used in this study. In addition, as no difference in arousal between positive and negative news was noted, it was ensured that the difference in EEG responses when listening to positive and negative news was not due to arousal.

Moreover, a significant main effect of News on interest (*F* (2, 266) = 22.91, *p* = 6.64 × 10^–10^, η_*p*_^2^ = 0.147) was found (Fig. 2c), revealing that the negative news showed significantly higher interest than the positive (*p* = 3.83 × 10^–4^) and neutral ones did (*p* = 2.12 × 10^–10^) and that the positive news was more interesting than neutral news was (*p* = 0.021).

Finally, a significant main effect of News on comprehensibility (*F* (2, 266) = 114.63, *p* = 1.25 × 10^–36^, η_*p*_^2^ = 0.463) was found (Fig. 2d), revealing that the positive news was significantly more comprehensible than the negative (*p* = 7.29 × 10^–10^) and neutral (*p* = 3.14 × 10^–28^) items and the negative news was more comprehensible than the neutral news (*p* = 7.37 × 10^–15^). In addition, a significant main effect of Group (*F* (1, 133) = 20.86, *p* = 1.11 × 10^–4^, η_*p*_^2^ = 0.136) was found: news was more comprehensible to the non-depressed individuals than to the depressed individuals. There was no interaction between News and Group for affective valence (*p* = 0.105), arousal (*p* = 0.758), interest (*p* = 0.258), or comprehensibility (*p* = 0.075).

### EEG responses while listening to news

**Figure 3:**
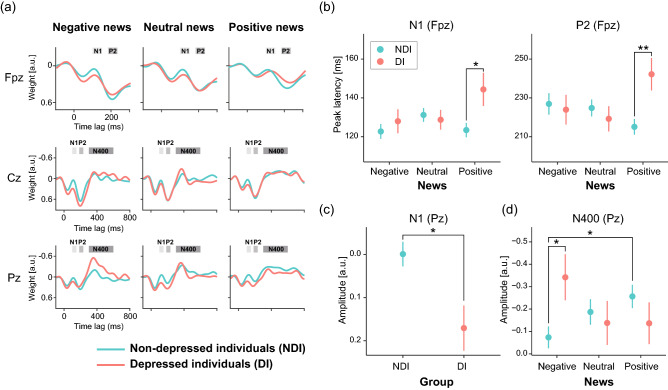
Differences in EEG between groups and between news contents. (**a**) The grand-averaged TRF waveforms in the depressed (pink) and non-depressed individuals (cyan) by channel and news are shown. The highlighted time windows were used to calculate the mean amplitudes of the components corresponding to N1, P2, and N400, respectively. (**b**) The depressed individuals had longer peak latencies of N1 and P2 at Fpz for the positive news than the non-depressed individuals. (**c**) The non-depressed individuals showed more negative deflection than the depressed individuals did in the N1 amplitude at Pz. (**d**) The depressed individuals showed more negative deflection in the N400 amplitude at Pz than the non-depressed individuals did while listening to the negative news. In addition, the negative news caused smaller negative deflection than the positive news, only in the non-depressed individuals. Each circle and error bar of (**b**)–(**d**) represent the mean and standard error, respectively. The depressed and non-depressed individuals are colored in pink and cyan, respectively. *p < 0.05 and **p < 0.01.

To investigate brain activity in response to each piece of news, we calculated TRF weights that describe the linear mapping between ongoing stimuli (words within news) and ongoing EEG data. The grand-averaged TRF waveforms clearly showed three components, corresponding to N1, P2, and N400 as reported in ERP studies (Fig. 3a). To clarify the effect of the group and/or news contents on these types of processing, we performed the two-way mixed-design ANOVA, using the between factor of Group and within factor of News, on the latency and amplitude of each component.

### Peak latencies

**Figure 4:**
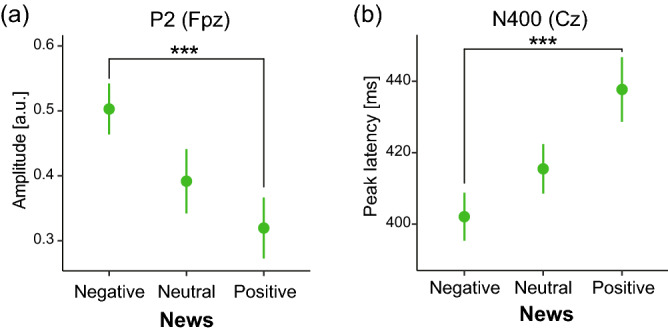
Main effect of News on EEG. (**a**) The P2 amplitude at the Fpz was larger for the negative news than for the positive news. (**b**) The peak latency for N400 for negative news at the Cz was shorter than that for positive news. Each circle and error bar represent the mean and standard error, respectively. ***p < 0.005.

Significant interactions, Group × News, were found for the peak latencies of N1 (*F* (2, 266) = 3.38, *p* = 0.036, η_*p*_^2^ = 0.025) and P2 (*F* (2, 266) = 4.78, *p* = 0.009, η_*p*_^2^ = 0.035) at the Fpz (Fig. 3b). This revealed that, compared with the non-depressed individuals, the depressed individuals had averages of 21 ms and 27 ms longer latencies of N1 (*p* = 0.034) and P2 (*p* = 5.79 × 10^–3^), respectively, in the positive news. In addition, the peak latency for N400 at the Cz showed a significant main effect of News (*F* (2, 266) = 4.42, *p* = 0.013, η_*p*_^2^ = 0.032) (Fig. 4b), which revealed that the latency for negative news was on average 33.3 ms shorter than that for positive news (*p* = 0.002).

### Amplitudes

The significant main effect of Group was found in the amplitudes of N1 at the Pz (*F* (1, 133) = 6.45, *p* = 0.012, η_*p*_^2^ = 0.046): the non-depressed individuals showed more negative deflection than the depressed individuals did (Fig. 3c). A significant main effect of News on the P2 amplitude (*F* (2, 266) = 7.62, *p* = 0.001, η_*p*_^2^ = 0.054) was found at the Fpz (Fig. 4a). This revealed that the negative news elicited a larger P2 than the positive news (*p* = 2.95 × 10^–4^).

Regarding amplitudes of N400, the significant interaction Group × News was found at Pz (*F* (2, 266) = 4.05, ε = 0.95, *p* = 0.020, η_*p*_^2^ = 0.030) (Fig. 3d). This revealed that the depressed individuals showed more negative deflection in the N400 amplitude than the non-depressed individuals did while listening to the negative news (*p* = 0.034). In addition, the negative news caused smaller negative deflection than the positive news, only in the non-depressed individuals (*p* = 0.017).

### Classification of depressed or non-depressed individuals

We evaluated the performances to classify depressed or non-depressed individuals, based on our subjective evaluation and EEG measures. Between the EEG and subjective evaluation data, for which the main effect of Group or the interaction of Group × News was significant in the ANOVA, we prepared three feature sets. These included subjective evaluation-only features (six features: arousal and comprehensibility for each news condition), EEG-only features (12 features: peak latencies of N1 and P2 at Fpz and mean amplitudes of N1 and N400 at Pz, for each news condition), and the Combination features of EEG and subjective evaluation (18 features). For classification, linear SVM was trained and evaluated using the leave-one-out cross-validation (LOOCV). The EEG-only and the Combination features were selected using the recursive-feature elimination method, and six features were used. To determine whether the performances of the classifiers trained using each feature were by chance, the permutation test was performed by randomizing the labels 1000 times, and the empirical *p*-values were calculated for each feature^47^.

**Figure 5:**
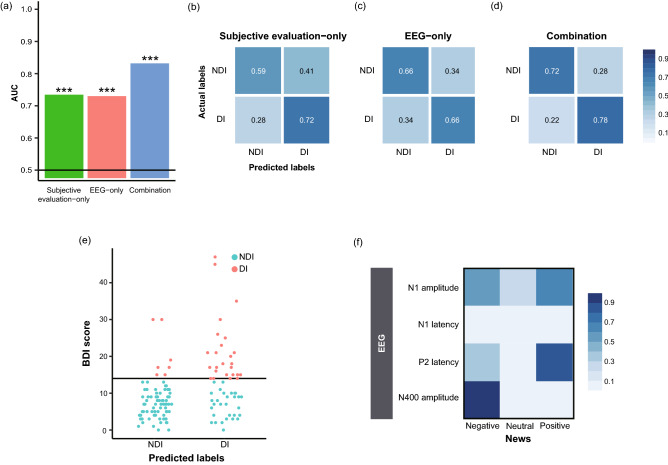
Classifications of individuals’ states based on the EEG and/or subjective evaluation. (**a**) AUC values of classification based on the Subjective evaluation-only features, EEG-only features, and the Combination features (***p < 0.005). The confusion matrix of classification results based on (**b**) subjective evaluation-only features, (**c**) EEG-only features, and (**d**) combination features. Since the matrix values represent the ratio of the number of predicted labels to the total number of participants in the non-depressed individuals (NDI) and depressed individuals (DI) for each row, the diagonal components of the confusion matrices represent true negative rate (TNR) and true positive rate (TPR), respectively. (**e**) Number of participants in the NDI and DI per predicted label; the y-axis represents the BDI-II score of the participant. (**f**) The mean absolute coefficients value of each feature in the EEG-only features. For visibility, the coefficient values were normalized to have a range of [0, 1].

The area under the receiver operating characteristic curves (AUCs) of the Subjective evaluation-only, EEG-only, and Combination features achieved 0.735, 0.730, and 0.832, respectively (Fig. 5a), indicating that all sets of the features can successfully discriminate depressed individuals. The permutation tests revealed that these performances of the classifiers were all significant (all *p*s = 0.001). The true positive rate (TPR), which is the rate of the depressed individuals, classified as the depressed individuals for the Subjective evaluation-only and EEG-only features was 0.719 (23/32 individuals) and 0.656 (21/32), respectively (Fig. 5b and c). The true negative rate (TNR), which is the rate of the non-depressed individuals, classified as the non-depressed individuals, was 0.592 (61/103) for the Subjective evaluation-only features and 0.660 (68/103) for the EEG-only feature. In the classification based on the Combination features, the TPR and TNR were 0.781 (25/32) and 0.718 (74/103), respectively (Fig. 5d). Figure 5e shows the number of depressed and non-depressed individuals per predicted label when using the Combination features.

The features’ contribution to the classification was analyzed by calculating the mean absolute values of the coefficients for the EEG-only features across all trained models (Fig. 5f), and the following six features were mainly selected: N1 amplitudes for negative, neutral, and positive news; P2 peak latencies for negative and positive news; and N400 amplitude for negative news. The feature with the highest coefficient values was the mean amplitude of N400 for negative news.

## Discussion

We tested two hypotheses to clarify whether attention to affective information differed between depressed and non-depressed individuals, and how attentional bias influences language processing. The first hypothesis states that negativity bias is stronger for depressed individuals than for non-depressed individuals. So far, previous behavioral studies’ results are inconsistent, with some studies confirming a greater attentional engagement to negative information than to positive information^33,48^ and difficulty in attentional disengagement from negative information^9,16,17^, while in individuals with subclinical depression^16,17^; others did not demonstrate such effects^24,25^. In the present study, we focused on the N1 and P2 components to test the hypothesis. We found that news contents had a significant effect on the amplitude of P2, but not N1, with its amplitude being significantly larger for negative news than for positive news. Given that the P2 amplitude is enhanced by attention^49^, which was confirmed especially when using affective stimulus^50–52^ in visual studies, our result shows more attentional resources were allocated to negative information than to positive information, while participants were listening to the news. The P2 amplitude did not show a significant main effect of Group, which suggests that contrary to the hypothesis, negativity biases were observed regardless of the depressed state. Furthermore, from the results that the peak latency of N400, which reflects semantic processing^41,42,44^, was shorter for negative news than for positive news in both groups, more attentional resources allocated to negative information could accelerate the subsequent semantic processing, regardless of the depressed state. To the best of our knowledge, this is a novel study on the effect of the attentional bias for negative information on the peak latency of semantic processing.

Other than negativity bias, delayed attention to positive information could be a characteristic of natural speech processing in depressed individuals: compared with the non-depressed individuals, the depressed individuals showed longer latencies in N1 and P2 to words within the positive news content. A previous ERP study using visual oddball paradigm reported a longer latency of early components (P1) to positive stimulus than to negative stimulus in individuals with major depression^53^. Compared with the controls, behavioral data also showed delayed attention to positive stimulus in patients with major depression^22^ and in individuals with subclinical depression and anxiety^17^. Consistent with these results, the present result suggests that the attention to inputs is delayed in depressed individuals more than in non-depressed individuals, while they listen to positive information.

The second hypothesis states that owing to the affective interference, the depressed individuals require more load on language comprehension (semantic processing on words) while listening to negative news, than the non-depressed individuals. The result supports that the depressed individuals showed a negatively larger amplitude of N400 during the negative news than the non-depressed individuals. This result is consistent with a previous ERP study that used the emotional Stroop paradigm, indicating that negative words produced a negatively larger amplitude of N400-like component in participants with major depression and remitted depression than in healthy participants^54^. These results support the affective interference theory^34^: owing to the allocation of attentional resources to negative information, depressed individuals required a greater load on non-affective processing (i.e., semantic processing). Our series of results suggest that depressed individuals are characterized by a spillover of the effects of attentional bias for negative information to other types of processing.

To detect depressed individuals, the EEG-only features reached an AUC of 0.730, which is a metric in which the closer a number is to 1, the better the performance (0.5 = random classification). Combining the subjective evaluation features with the EEG features improved performance, and the highest performance of 0.832 in AUC was achieved. These classification performances highlight the effectiveness of EEG measures in successfully detecting depressed individuals. Previous studies on EEG-based depression patient detection have focused on resting-state data, using features such as power values and functional connectivity metrics across channels^55,56^. In contrast, the current study focused on detecting depressed individuals based on their daily activities, and, for the first time, it demonstrates the feasibility of the EEG measured while listening to news.

The detection method used in this study was advantageous in that the cognitive function of the EEG features used was more clearly interpretable based on a huge number of previous ERP studies. The coefficient values of the EEG-only features indicated that the N400 amplitude to words within negative news contributed the most to the classification performance, suggesting that EEG features related to the semantic processing of natural speech are involved in the detection of depressed individuals. The contribution of EEG features in processing negative information is consistent with previous results that, the best classification performance for detecting individuals with mild depression in a non-clinical population was obtained when EEG features were used during the presentation of negative face stimuli^57^. In addition, the N1 amplitude and P2 peak latency—modulated by attention to the stimuli^38^—mainly for affective news contents (N1 amplitude: negative, neutral, and positive news, P2 peak latency: negative and positive news, cf. Fig. 5f) were also contributed to classification performance. These results suggest that individuals who increase the allocation of attentional resources and semantic processing load to affective speech stimuli are likely to be classified as having depression. In recent years, technologies to monitor cognitive or mental states such as cognitive load^58^ and motivation^59^ using brain activities under real-world conditions or situations close to the real have been widely studied. Consistent with this trend, we envision an application based on EEG that notifies individuals of their depressed state as predicted by their EEG so that it could lead to changing their behavior for using information, such as spending less time exposed to negative information on the Internet and social media. The promising classification performances in this study using natural speech as stimuli would lead to the development of such an application based on EEG and encourage individuals to change the way they use information according to their state of depression. In reality, the feasibility of such an application is high because a depressed state can be detected simply by listening to news auditory clips. In the future, it will be necessary to test whether providing feedback on the depressed state actually promotes such behavioral changes in individuals.

As a limitation, since the study ultimately aimed to develop an application based on EEG that provides feedback about individuals’ states of depression in daily life rather than to detect or diagnose clinical depression, we recruited people who had not been diagnosed with any mental disease in the study; depressed and non-depressed individuals were determined based solely on their BDI-II scores. Therefore, the depressed individuals in this study may have included those with clinical depression. It cannot be denied that there are differences in EEG responses between individuals with clinical depression and those who have not been diagnosed with depression, which may affect results obtained in this study. Future research will address this issue.

## Conclusion

This study found that depressed individuals are characterized by the delayed attention to positive news and a higher semantic processing load for negative news. The classification model trained by these EEG features successfully classified depressed and non-depressed individuals, suggesting that EEG measures during daily life activities, such as listening to news items, provide a way to ascertain the depressed state.

## Methods

### Participants

Participants in the EEG experiment included 162 adult native Japanese speakers (88 women and 74 men, *M*_age_ = 34.2, *SD*_age_ = 10.1, range_age_ = 20–49). All participants had normal hearing and normal or corrected-to-normal vision. They had no history of neurological or psychiatric diseases. The data sets for participants whose EEG data contained large artifacts were excluded from the analysis; finally, data for 135 participants were used (69 women and 66 men, *M*_age_ = 33.2, *SD*_age_ = 10.1, range_age_ = 20–49). The participants’ handedness was estimated based on the laterality quotient (LQ) of the Edinburgh Handedness Inventory^60^. Of the participants, 124 were classified as right-handed (*M*_LQ_ = 92.5, *SD*_LQ_ = 12.1), four as ambidextrous (*M*_LQ_ = − 18.2, *SD*_LQ_ = 24.6), and 7 as left-handed (*M*_LQ_ = − 83.0, *SD*_LQ_ = 18.0).

The participants’ depressed state was assessed using the BDI-II for Japanese^37^ before data collection. The questionnaire comprised 21 questions regarding the participants’ depressed state in the past two weeks, including the day of the experiment. There was no significant gender difference in the BDI-II scores between women (*M* = 10.13, *SD* = 7.77) and men (*M* = 9.98, *SD* = 8.28) by an independent samples *t*-test (*t* (133) = 0.12, *p* = 0.908, Cohen’s *d* = 0.020). The BDI-II guidelines state that scoring 14 or higher is the threshold at which individuals are classified as having depression. As such, 32 participants were classified in the depressed individuals (*M*_age_ = 31.3, *SD*_age_ = 10.5, range_age_ = 20–49, *M*_LQ_ = 70.1, *SD*_LQ_ = 55.6) and 103 in the non-depressed individuals (*M*_age_ = 33.8, *SD*_age_ = 10.0, range_age_ = 20–49, *M*_LQ_ = 82.8, *SD*_LQ_ = 40.2) based on their BDI-II scores. The participants’ characteristics are summarized in Table 1.

The participants’ academic history was as follows: 1 junior high school graduate, 18 high school graduates, 19 junior college or vocational school students or graduates, 85 university students or graduates, and 12 graduate students, master’s, or doctoral degree holders. To confirm the relationship between academic history and the participants’ group (depressed or non-depressed), a chi-square test of independence was performed. No significant difference was confirmed (${\chi }^{2}$(4) = 5.54, *p* = 0.246, Cramer's *V* = 0.203).

This study was approved by the Ethics Committee for Human and Animal Research of the National Institute of Information and Communications Technology and was carried out in accordance with The Code of Ethics of the World Medical Association (Declaration of Helsinki). Written informed consent to participate in this study was obtained from all participants.

### Materials

We prepared 30 news items from Japanese radio news programs (NHK Radio News; https://www.nhk.or.jp/radionews/) broadcasted by the Japan Broadcasting Corporation (NHK) in February 2019. Approximately one minute of the audio from the beginning of each news item was cut out for use as stimuli (ranged from 45 to 83 s).

To select positive, neutral, or negative news to be used in the EEG analysis from those 30 news items, we conducted a preliminary survey through subjective evaluations of 160 native Japanese speakers (82 women and 78 men, *M*_age_ = 35.9, *SD*_age_ = 8.4, range_age_ = 20–49) who did not participate in the EEG experiment. We selected each of five news items with (1) significant differences in affective valence among negative, neutral, and positive news items and (2) no significant differences in arousal among positive and negative news items. This was done as follows: participants read each news item and then subjectively evaluated the affective valence (1: negative–5: positive), arousal (1: low arousal–5: high arousal), interest (1: not interest at all–5: high interest), and comprehensibility (1: very difficult–5: very easy) on a 5-point scale. Based on this evaluation, we selected five news items with high (3.6 or more), moderate (3.0–3.4), and low (2.2 or less) affective valence, to be used as positive, neutral, and negative news conditions. The topics of the selected news are shown in Fig. 1.

To confirm whether the subjective evaluation in the preliminary survey differed across news conditions, the subjective evaluation ratings were subjected to one-way repeated measures ANOVA. As the subjective evaluation values ranged from 1 to 5 and were non-normally distributed, an aligned rank transformation of the subjective evaluation values was performed using the ARTool^61,62^. To control for familywise error rates in multiple comparisons, *p*-values were adjusted based on the Bonferroni correction. The ANOVA results showed significant differences across news conditions in all items: the affective valence (*F* (1, 318) = 447.17, ε = 0.81, *p* = 7.87 × 10^–76^, η_*p*_^2^ = 0.738), arousal (*F* (1, 318) = 31.34, ε = 0.93, *p* = 2.11 × 10^–12^, η_*p*_^2^ = 0.165), interest (*F* (1, 318) = 25.95, *p* = 3.62 × 10^–11^, η_*p*_^2^ = 0.140), and comprehensibility (*F* (1, 318) = 66.91, *p* = 5.60 × 10^–25^, η_*p*_^2^ = 0.296). The affective valence of positive news was significantly higher than that of neutral and negative ones, and the affective valence of neutral news was significantly higher than that of negative news. Arousal due to both negative and positive news was significantly higher than that due to neutral news. The interest in negative news was significantly higher than that in neutral and positive ones, and positive news was significantly more than a neutral one. The comprehensibility of positive news was significantly higher than that of neutral and negative ones, and negative news was significantly more than that of a neutral one. All post-hoc test results for each item are summarized in Table 2. The differences in the ratings between the preliminary survey and EEG experiment are presented in Supplementary Table S2.

The EEG responses, time-locked to each word onset included in each news item, were analyzed. A Japanese native speaker identified the time of onset of each content word in each news item by listening to the audio and visually inspecting the sound waveform and spectrograms using WaveSurfer (https://sourceforge.net/projects/wavesurfer/), an open-source speech and acoustic analysis tool.

### EEG experimental procedure

The participants sat in chairs, in front of a monitor display and numeric keypad. In the EEG experiment, they listened to 30 news items (15 selected news in total; the remaining 15 news were included in the stimuli set and were used for other research purposes) and evaluated them subjectively. In each trial, the participants started a trial by pressing a key “1” on the numeric keypad, and immediately after, they listened to an audio clip of one news item binaurally using earphones (RHA Technologies Ltd., United Kingdom). To suppress their eye movements, participants were instructed to gaze at a fixation point (+) presented at the center of the display while listening to the news. After each news item, participants were asked to subjectively evaluate the affective valence, arousal, interest, and comprehensibility of the news, based on a 5-point scale. The procedure for the subjective evaluation was the same as in the preliminary survey. Participants performed a total of six blocks (five trials/block). The data collection lasted approximately 1 h.

EEG and electrooculogram (EOG) signals were continuously measured throughout all blocks using an eight-channel wireless EEG device and measurement software (Polymate Mini AP108 and Mobile Acquisition Monitor 2.02, Miyuki Giken Co. Ltd., Japan). Because this study envisioned an application based on EEG that could be used in real-world environments, we focused on a few electrodes that were relatively easy to place rather than a high-density electrode system. Since N1/P2 and N400 to the auditory stimuli are dominantly observed in the fronto-central regions^63^ and centro-parietal regions^64^, respectively, active electrodes were placed on Fpz, Cz, and Pz locations, according to the International 10–10 system for EEG measurement. The Fpz location was chosen instead of Fz because it is easier to place the electrode on the forehead, where interference by hair can be avoided. Previous research has demonstrated that the Fpz electrode can successfully observe the N1 and P2 components to auditory stimuli^65^. Two electrodes were placed on the lateral of the left outer canthus and above the left eye to measure horizontal and vertical EOGs, respectively. All signals were sampled at 500 Hz using reference and ground electrodes placed at the right and left earlobes, respectively.

### EEG analysis

MATLAB (MathWorks Inc., USA) and EEGLAB toolbox^66^ were used to preprocess EEG signals. A band-pass finite impulse response (FIR) filter between 1 and 50 Hz (3300th order) was applied to the signals, and it was resampled at 200 Hz. The transient, large-amplitude artifacts were removed by the artifact subspace reconstruction^67,68^. Artifacts of eye blink and eye movements were excluded from the data using an independent component analysis. Finally, a further band-pass FIR filter of 1–8 Hz (1320th order) was applied to the signals to improve the signal-to-noise ratio.

To estimate the TRFs that describe the linear mapping between a stimulus and the preprocessed EEG data for each channel, we prepared a stimulus matrix at the same sampling rate as the preprocessed EEG data for each news item. The stimulus matrix comprised time-aligned impulses with a value of 1 at the content word onset time points and 0 at other time points. We used the mTRF toolbox in MATLAB^45^ for the estimation. The continuous EEG response ${r}_{ch}\left(t\right)$ at a time point *t* was assumed to comprise convolutions of the stimulus vector *s*(*t*) and TRF weights ${w}_{ch}$($\tau$) at *ch*-th channel:

1 $$r_{ch} \left( t \right) = \mathop \sum \limits_{\tau } w_{ch} \left( \tau \right)s\left( {t - \tau } \right) + \varepsilon_{ch} \left( t \right),$$

where ε_*ch*_(*t*) is the residual response in the *ch*-th channel. Ridge regression, with a regularization parameter λ, was used to estimate the TRF weights over a range of time lag τ from − 100 to 800 ms, relative to each stimulus onset. Using the mTRFcrossval function^45^, we estimated the optimal regularization parameters for each participant based on the LOOCV, in which one trial is evaluated as test data and the remaining trials as training data, and the evaluation is repeated until all trials are used as test data. In this procedure, the following steps were applied to each participant’s channel data: (1) the TRF weights were estimated for each single-trial data, (2) the single-trial weights were averaged over the training data, and (3) the mean squared errors (MSEs) between the actual responses and the responses predicted using the averaged weights were computed using the test data. In the range [2^1^–2^21^], for each participant, we determined the optimal regularization parameter λ that resulted in the smallest MSEs, averaged over the test data and the channel. Finally, the regularization parameter, λ = 2^13^, which was the most frequent value in the distribution of regularization parameters estimated by each participant (*N* = 42), was adopted for all participants. Finally, the single-trial TRF weights for each participant, estimated using the regularization parameter, were averaged by news condition per channel.

For each channel, the mean amplitude per component of a single participant’s TRF weight for each news condition was calculated using time windows of [100, 160 ms], [170, 230 ms], and [300, 600 ms] for N1, P2, and N400, respectively. These time windows were determined through the visual inspection of grand-averaged TRF weights. The peak latency of each component was also detected per channel in each news condition. To detect peak latencies, we used a dynamic time warping (DTW) algorithm, which is a technique used to non-linearly map two temporal sequences that vary in time or speed^69^. DTW has been used to automatically detect peak latencies of ERP components^70,71^. First, we calculated the grand-averaged TRF waveforms for all participants and each news condition for each channel, from which we detected the peak latencies for the three components. Thereafter, for each channel, the TRF for each participant and the grand-averaged TRF were mapped onto a common time axis using DTW, as such, the time point corresponding to the peak latency obtained from the grand-averaged TRF was automatically identified.

### Statistical analysis

To analyze subjective evaluation, a two-way mixed-design ANOVA was employed to analyze whether subjective evaluation was affected by News (within-subjects factor: negative, neutral, and positive) and Group (between-subjects factor: depressed individuals and non-depressed individuals). For analysis using ANOVA, each subjective evaluation was performed on an aligned rank transformation as well as the preliminary survey. To investigate the effect of Group and News on the amplitude or peak latency of each component corresponding to N1, P2, and N400, a two-way mixed-design ANOVA was conducted. When the main effect of News was statistically significant, multiple comparisons of paired *t*-tests across news conditions were conducted. When significant interaction of Group × News was obtained, multiple comparisons of paired *t*-tests across news conditions were conducted for each group. In addition, to investigate the simple main effects of Group, unpaired *t*-tests between the groups were administered per the new condition.

In all statistical test procedures, the significance level was set at 0.05. If Mauchly’s test showed that homogeneity of variance was violated, the degree of freedom was adjusted using the Greenhouse–Geisser procedure. When the degree of freedom was adjusted using the procedure, the original degree of freedom and ε were reported. We report partial eta squared as an effect size. For all multiple comparisons, *p*-values were adjusted using the Bonferroni procedure.

### Classification with a linear SVM

To investigate whether EEG measurements play a role in the detection of depressed individuals, we performed a binary classification of depressed and non-depressed individuals based on the EEG features and/or subjective evaluation of the news. As features for classification, we used explanatory variables showing the significant main effect of Group or interaction of Group × News in the ANOVA: 12 EEG features (peak latencies of N1 and P2 at Fpz and mean amplitudes of N1 and N400 at Pz, for each news item) and 6 subjective evaluation features (arousal and comprehensibility for each news item). To compare the results, based on the features used, three sets of features were prepared: 12 features of only EEG, 6 features of only subjective evaluation, and 18 features combining EEG and subjective evaluation (hereinafter referred to as the EEG-only features, Subjective evaluation-only features, and Combination features, respectively). The feature vectors were standardized per feature by subtracting the mean value and then dividing the difference by the SD. The mean values and SD were calculated using the training data. For classification, we used a linear SVM, which is widely used in the Brain-Computer Interface context because of the robustness to outliers, due to the regularization and applicability to high-dimensional data sets^72^, with the squared hinge loss and the L2 penalty.

The performance of the linear SVM was evaluated using the LOOCV, whereby one participant’s data was reserved for a test and the remaining data were used for model training. In the LOOCV, the procedure was repeated so that every participant’s data was evaluated as test data once. For the EEG-only features and the Combination features, the feature selection procedure involved the recursive-feature elimination method. In each LOOCV procedure, six features were selected based on the coefficients of the linear SVM trained on the training data, and the selected features were used for model training and evaluation. A cost parameter *C,* which is a hyperparameter that determines the degree of tolerance for classification errors in the training data, was optimized using the grid-search. The best performing cost parameter in the stratified threefold cross-validation of the training data was selected from [*C* = 10^–3^, 10^–2^, 10^–1^, 10^0^, 10^1^, 10^2^, 10^3^].

The number of data samples per class was imbalanced (non-depressed individuals: *N* = 103, depressed individuals: *N* = 32). Therefore, the costs of the classification errors were adjusted per class by multiplying the cost parameter *C* by the weights, based on the number of training samples in the class (i.e., the total number of samples/(the number of classes × the number of samples in the class)). Another widely used approach to handle imbalanced data is resampling, which artificially balances the class distribution of the training data by over- (i.e., increasing the number of samples belonging to the minority class) and/or under-sampling (i.e., removing samples belonging to the majority class). The classification performance was evaluated using AUC and those using the approach are provided in Supplementary Fig. S1. The permutation test was conducted to determine whether the classification performances using each future were by chance. In the test, a null distribution was constructed under the null hypothesis that there was no dependence between the features and the labels on each other by randomizing the labels, an then calculating an empirical *p*-value of the obtained AUC against that null distribution, which is the proportion of the randomized labels that performs as well or better than the original label^47^. The total number of randomizations was set to 1000.

To clarify which features contributed to the classification performances, among the features that showed differences in EEG responses to affective news depending on individuals’ depressed state, we computed the absolute values of the coefficients assigned to each feature of the models trained by the EEG-only features. As LOOCV repeats the training and evaluation of the same number of data samples, the values of the coefficients were averaged over all models trained in LOOCV. If a feature was not selected in the feature selection procedure applied to the EEG-only features, its coefficient was treated as having zero value in this analysis. The scikit-learn library for Python was used for the classification procedures^73^.

## Supplementary Information

Supplementary Information.

## Data Availability

The data set presented in this research is partially available upon request to the corresponding author, limited to the data that the participant has agreed to make publicly available.
