# Supplementary material for: Impact of depressed state on attention and language processing during news broadcasts: EEG analysis and machine learning approach
Source: Sci Rep. 2022 Nov 28;12:20492. doi: 10.1038/s41598-022-24319-x (PMC9703439; doi:10.1038/s41598-022-24319-x)
Supplement: Supplementary file 1 — Supplementary Information. [file 41598_2022_24319_MOESM1_ESM.pdf]

Supplementary Information

Impact of depressed state on attention and language processing during news broadcasts: EEG analysis and machine learning approach

Authors:

Kohei Fuseda[†, ‡], Hiroki Watanabe[†], Atsushi Matsumoto[‡], Junpei Saito, Yasushi Naruse, and Aya S. Ihara*

†These authors have contributed equally to this work and share the first authorship.

Affiliations:

Center for Information and Neural Networks, Advanced ICT Research Institute, National Institute of Information and Communications Technology, and Osaka University, 588-2 Iwaoka, Iwaoka-cho, Nishi-ku, Kobe, Japan

‡Present address

Kohei Fuseda, Bunkyo Gakuin University

Atsushi Matsumoto, Kansai University of Welfare Sciences

***Corresponding author:** Aya S. Ihara (ihara@nict.go.jp)

Supplementary Table S1. Citations of news used in the experiment

News	Citations
Negative	NHK Radio News at 5 p.m. (Feb. 16, 2019)
	NHK Radio News at 10 a.m. (Feb. 19, 2019)
	NHK Radio News at noon (Feb. 20, 2019)
	NHK Radio News at noon (Feb. 20, 2019)
	NHK Radio News at 8 p.m. (Feb. 17, 2019)
Neutral	NHK Radio News at 9 p.m. (Feb. 19, 2019)
	NHK Radio News at 9 p.m. (Feb. 16, 2019)
	NHK Radio News at 8 a.m. (Feb. 19, 2019)
	NHK Radio News at 6 a.m. (Feb. 19, 2019)
	NHK Radio News at 3 p.m. (Feb. 16, 2019)
Positive	NHK Radio News at 3 p.m. (Feb. 19, 2019)
	NHK Radio News at 8 p.m. (Feb. 17, 2019)
	NHK Radio News at 7 p.m. (Feb. 16, 2019)
	NHK Radio News at 2 p.m. (Feb. 17, 2019)
	NHK Radio News at 9 a.m. (Feb. 19, 2019)

Supplementary Table S2. Subjective rating to the negative, neutral, and positive news in the preliminary survey (reading) and EEG experiment (listening)

Items	Preliminary survey (N = 160)			EEG experiment (N =135)		
	Negative	Neutral	Positive	Negative	Neutral	Positive
Affective valence	1.8 ± 0.6	3.2 ± 0.5	3.9 ± 0.7	1.6 ± 0.5	3.3 ± 0.5	4.2 ± 0.6
Arousal	3.1 ± 0.7	2.8 ± 0.6	3.0 ± 0.7	3.4 ± 0.7	2.8 ± 0.6	3.3 ± 0.7
Interest	3.1 ± 0.8	2.7 ± 0.7	2.8 ± 0.8	3.6 ± 0.7	3.0 ± 0.7	3.3 ± 0.8
Comprehensibility	3.7 ± 0.8	3.4 ± 0.7	3.9 ± 0.7	4.0 ± 0.8	3.5 ± 0.7	4.4 ± 0.6

Mean ± SD.

Each piece of news was visually presented to the participants in the preliminary survey and was auditorily presented in the EEG experiment. Therefore, the subjective evaluation could differ between reading (preliminary survey) and listening (EEG experiment). To clarify whether the subjective ratings were influenced by differences in the modality, a two-way mixed-design ANOVA was employed to analyze whether (the ART transformed) subjective evaluation was affected by News (within-subjects factor: negative, neutral, and positive) and Modality (between-subjects factor: reading [preliminary survey] and listening [EEG experiment]).

Affective valence

A significant interaction, News × Modality, was found (F (2, 690) = 25.16, ε = 0.98, $p = 4.25\times10^{-11}$, $\eta_p^2 = 0.068$). The ratings for the positive ($p = 1.72\times10^{-10}$) and neutral ($p = 0.02$) news when listening was significantly higher than that when reading. On the other hand, the rating when listening to negative news was significantly lower than when reading negative news ($p = 1.72\times10^{-8}$). The order of affective valence was common for both modalities: negative news < neutral news (reading: $p = 1.25\times10^{-57}$, listening: $p = 3.58\times10^{-70}$) and neutral news < positive news (reading: $p = 3.48\times10^{-32}$, listening: $p = 1.84\times10^{-42}$).

Arousal

A significant interaction, News × Modality, was found (F (2, 690) = 9.65, ε = 0.97, $p = 1.08\times10^{-4}$, $\eta_p^2 = 0.027$). The ratings for the negative ($p = 3.58\times10^{-4}$) and positive news ($p = 5.46\times10^{-4}$) when listening was significantly higher than when reading. In both modalities, the ratings for the negative news (reading: $p = 3.08\times10^{-12}$, listening: $p = 3.58\times10^{-19}$) and positive news (reading: $p = 1.28\times10^{-7}$, listening: $p = 7.49\times10^{-16}$) were significantly higher than that for the neutral news. In contrast, the ratings for the negative news and the positive news showed no significant difference (reading: $p = 0.07$, listening: $p = 0.19$).

Interest

A significant interaction, News × Modality, was found (F (2, 690) = 3.80, p = 0.02, $\eta_p^2 = 0.011$). The rating when reading was significantly higher for the negative news than for the positive ($p = 8.00\times10^{-6}$) and neutral ones ($p = 3.86\times10^{-11}$). The ratings when listening was significantly higher for the negative news than for the positive ($p = 1.10\times10^{-5}$) and neutral ones ($p = 1.52\times10^{-16}$), and higher for the positive news than for the neutral one ($p = 6.55\times10^{-4}$). In addition, the ratings when listening were significantly higher than when reading (negative: $p = 5.73\times10^{-12}$, positive: $p = 4.76\times10^{-9}$, neutral: $p = 1.00\times10^{-5}$).

Comprehensibility

A significant interaction, News × Modality, was found (F (2, 690) = 23.93, ε = 0.98, $p = 1.38\times10^{-10}$, $\eta_p^2 = 0.065$). In both modalities, the rating was significantly higher for the positive news than that for the neutral news (reading: $p = 1.09\times10^{-22}$, listening: $p = 3.19\times10^{-42}$) and negative ones (reading: $p = 4.65\times10^{-7}$, listening: $p = 1.39\times10^{-12}$), and also higher for the negative news than that for the neutral news (reading: $p = 1.84\times10^{-9}$, listening: $p = 6.72\times10^{-20}$). In addition, the ratings when listening were significantly higher than those when reading (negative: $p = 2.00\times10^{-6}$, positive: $p = 6.09\times10^{-12}$, neutral: $p = 0.03$).

To summarize the results, independent of modality, affective valence was higher for the positive, neutral, and negative, in that order, and arousal did not differ between the positive and negative as we supposed. This indicates that the selection of the news used in the EEG experiment was appropriate so that we could discuss the difference in the processing of affective information between depressed and non-depressed individuals.

However, we found interesting differences by modality. First, affective valence was higher for the positive and neutral news (i.e., more positive) and lower for the negative news (i.e., more negative) when listening compared to reading. Second, arousal was higher for the negative and positive news when listening compared to reading. Third, interest and comprehensibility were higher when listening than when reading. When listening to the news, participants received not only verbal information but also nonverbal information such as emotional prosody, unlike when reading. Therefore, this difference in subjective evaluation when listening from when reading may be caused by the presence of nonverbal information.

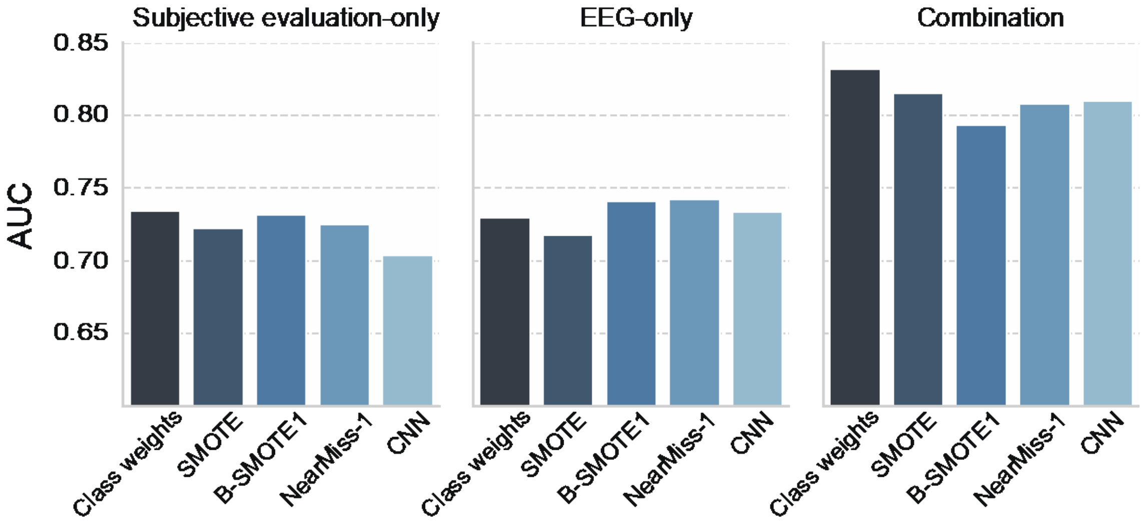

Supplementary Fig. S1. Performance comparison between resampling methods to handle the imbalanced data. The classification performances when the resampling methods were applied for the Subjective evaluation-only features, EEG-only features and Combination features are provided. For oversampling, we used the Synthetic Minority Over-sampling TEchnique (SMOTE)[1] and borderline-SMOTE1 (B-SMOTE1)[2]—the derivative of SMOTE. For under-sampling, we used the NearMiss-1[3] and the Condensed Nearest Neighbor rules (CNN)[4]. The python toolbox *Imbalanced-learn*[5] was employed to implement these methods. The classification reported in the main texts used the class weights to handle the imbalanced data (Class weights).

References

1 Chawla, N. V., Bowyer, K. W., Hall, L. O. & Kegelmeyer, W. P. SMOTE: synthetic minority over-sampling technique. *J. Artif. Intell. Res.* **16**, 321-357, http://dx.doi.org/10.1613/jair.953 (2002).

2 Han, H., Wang, W. Y. & Mao, B. H. Borderline-SMOTE: a new over-sampling method in imbalanced data sets learning. in *Proc. Int. Conf. Intelligent Computing* 878-887 (2005).

3 Zhang, I. & Mani, I. kNN approach to unbalanced data distributions: a case study involving information extraction. in *Proc. Workshop on Learning from Imbalanced Data Sets* 1-7 (2003).

4 Hart, P. The condensed nearest neighbor rule (corresp.). *IEEE Trans. Inf Theory* **14**, 515-516 (1968).

5 Lemaître, G., Nogueira, F. & Aridas, C. K. Imbalanced-learn: A python toolbox to tackle the curse of imbalanced datasets in machine learning. *J. Mach. Learn. Res.* **18**, 1-5, https://dl.acm.org/doi/10.5555/3122009.3122026 (2017).
